# Osteomodulin modulates the inflammatory responses via the interleukin-1 receptor 1/nuclear factor-κB signaling pathway in dental pulpitis

**DOI:** 10.1038/s41368-025-00369-5

**Published:** 2025-05-26

**Authors:** Yueyi Yang, Xuchen Hu, Meiling Jing, Xiaohan Zhu, Xiaoyu Liu, Wenduo Tan, Zhanyi Chen, Chenguang Niu, Zhengwei Huang

**Affiliations:** 1 Department of Endodontics, Shanghai Ninth People’s Hospital, Shanghai Jiao Tong University School of Medicine, College of Stomatology, Shanghai Jiao Tong University, Shanghai, China; 2 National Clinical Research Center for Oral Diseases, National Center for Stomatology, Shanghai Key Laboratory of Stomatology, Shanghai, China

**Keywords:** Regeneration, Nuclear receptors

## Abstract

Pulpitis is a common infective oral disease in clinical situations. The regulatory mechanisms of immune defense in pulpitis are still being investigated. Osteomodulin (OMD) is a small leucine-rich proteoglycan family member distributed in bones and teeth. It is a bioactive protein that promotes osteogenesis and suppresses the apoptosis of human dental pulp stem cells (hDPSCs). In this study, the role of OMD in pulpitis and the OMD-induced regulatory mechanism were investigated. The OMD expression in normal and inflamed human pulp tissues was detected via immunofluorescence staining. Intriguingly, the OMD expression decreased in the inflammatory infiltration area of pulpitis specimens. The cellular experiments demonstrated that recombined human OMD could resist the detrimental effects of lipopolysaccharide (LPS)-induced inflammation. A conditional *Omd* knockout mouse model with pulpal inflammation was established. LPS-induced inflammatory impairment significantly increased in conditional *Omd* knockout mice, whereas OMD administration exhibited a protective effect against pulpitis. Mechanistically, the transcriptome alterations of OMD overexpression showed significant enrichment in the nuclear factor-κB (NF-κB) signaling pathway. Interleukin-1 receptor 1 (IL1R1), a vital membrane receptor activating the NF-κB pathway, was significantly downregulated in OMD-overexpressing hDPSCs. Additionally, the interaction between OMD and IL1R1 was verified using co-immunoprecipitation and molecular docking. In vivo, excessive pulpal inflammation in *Omd*-deficient mice was rescued using an IL1R antagonist. Overall, OMD played a protective role in the inflammatory response via the IL1R1/NF-κB signaling pathway. OMD may optimize the immunomodulatory functions of hDPSCs and can be used for regenerative endodontics.

## Introduction

Pulpitis is a common infective disease mainly caused by the invasion of oral bacteria. The bacteria invade the tooth and damage the dentin and pulpal tissues, resulting in uncontrolled inflammation leading to pulpal destruction and hence severe pain.^1^ Therefore, limiting the inflammatory progression and preserving pulp activity are of great importance in the clinical management of pulpitis.

Human dental pulp stem cells (hDPSCs) are a type of stem cells enriched in pulp tissue possessing a multidirectional differentiation potential and immunophenotype of cellular self-defense.^2,3^ When a pulpal injury is caused by a bacterial infection or trauma, hDPSCs migrate toward the infected site and differentiate into odontoblasts, contributing to tissue regeneration.^4,5^ The outcome of pulpitis depends on the balance between regeneration and inflammation. Modulated inflammation seems to be a prerequisite for pulp repair, whereas excessive and uncontrolled inflammation can aggravate the destruction of pulp tissues and lead to the loss of dental function.^6,7^ The inflammatory response is characterized by the secretion of cytokines, such as interleukin-1β (IL-1β) and tumor necrosis factor-alpha (TNFα).^8,9^ Both IL-1β and TNFα are crucial for mediating the initial stage of the inflammatory process.^10,11^ The activation of nucleotide-binding oligomerization domain-like receptor protein 3 (NLRP3) inflammasome plays an important role in IL-1β maturation. During progressive pulpitis, the excessive secretion of proinflammatory cytokines can disturb the biological behaviors of hDPSCs, whereas the dysfunction of hDPSCs contributes to the pathological process of pulpitis.^12,13^ Hence, deciphering the immunoregulatory network of hDPSCs may be a potential strategy for treating inflamed dental pulp.

The extracellular matrix (ECM) molecules serve as an essential regulator in pulpal tissue repair, interacting with matrix molecules or membrane receptors and directing downstream signaling.^14^ Osteomodulin (OMD), also termed as osteoadherin, is a member of small leucine-rich proteoglycans (SLRPs), which are biologically active components mainly distributed in ECM.^15,16^ OMD is characterized by its high hydroxyapatite crystal-binding capacity and specific distribution in mineralized tissues, including bone and teeth. The OMD expression in pulp was identified in odontoblasts and pulpal fibroblasts.^17^ OMD serves as a critical mediator in pulp biology. Specifically, it is involved in the dental mineralization process by promoting odontoblastic differentiation of dental pulp cells. Also, it directly regulates the diameter and shape of type I collagen fibrils to organize the extracellular matrix.^18–20^ Our previous studies have demonstrated that OMD promotes the osteoblastic differentiation of hDPSCs by interacting with bone morphogenetic protein 2 (BMP2) and plays a protective role in the apoptosis of hDPSCs.^21,22^ Therefore, OMD is likely a crucial modulator in the bioactivity of hDPSCs. Increasing evidence has indicated a potential role of OMD in the inflammatory process. The proteomic analysis of human craniosynostosis revealed the upregulation of OMD in fused sutures. Further proteomic network analysis indicated that OMD was associated with IL-10 and IL-1β, both of which are key cytokines involved in inflammation and osteoclastogenesis.^23,24^ Additionally, OMD levels were found to be significantly downregulated in the osteoarthritic (OA) labrum group compared with the healthy group. This reduction was driven by excessive IL-1β secretion.^25^ Mechanistically, OMD regulated the inflammatory response in OA by directly binding to the complement inhibitor C4b-binding protein, thereby restricting excessive complement activation.^26^ These findings suggest that OMD is involved in the immunoregulation of some inflammatory diseases. As such, we speculated that OMD may be a candidate mediator resisting the inflammatory process in the infected pulp tissues.

This study aimed to investigate the involvement of OMD in the pathogenic process of dental pulpitis and decipher its underlying mechanism. OMD showed a decreased profile in pulpitis specimens. Lipopolysaccharide (LPS) was introduced into hDPSCs to mimic the pulpal inflammation in vitro. The findings revealed an inhibitory effect of OMD on the inflammatory response through the interleukin-1 receptor 1 (IL1R1)/nuclear factor-κB (NF-κB) signaling pathway. Furthermore, we constructed an experimental pulpitis mouse model in the conditional *Omd* knockout mice to investigate the inflammatory alterations upon OMD deficiency and ascertain the regulatory role of OMD. The optimized immunomodulatory functions of OMD in hDPSCs may serve as a promising target for regenerative endodontics.

## Results

### Decreased OMD expression in inflamed pulp specimens and LPS-induced hDPSCs

**Fig. 1:**
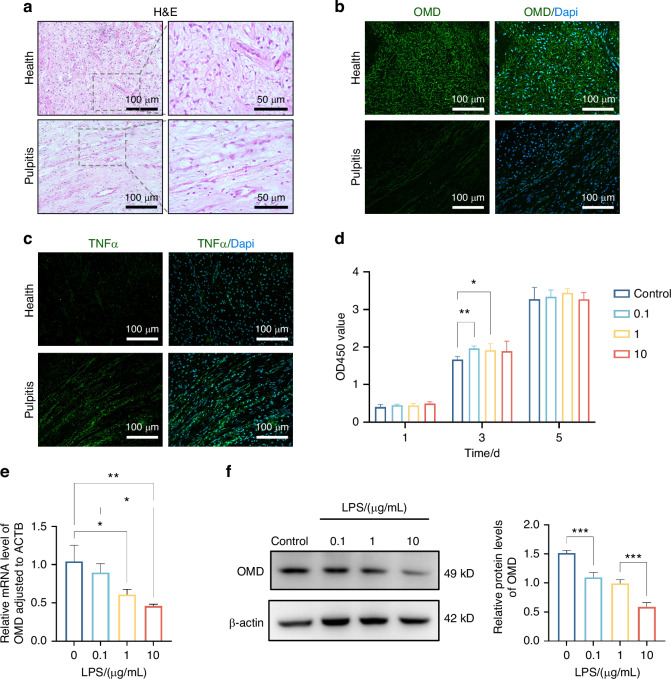
Decreased expression of OMD in pulpitis specimens and LPS-induced hDPSCs. **a**–**c** HE and immunofluorescence staining of healthy and inflamed pulpal tissues. **d** CCK8 assay results of hDPSCs after LPS treatment (0, 0.1, 1.0, and 10 μg/mL) for 1, 3, and 5 days. **e** mRNA expression of OMD in LPS-treated hDPSCs. **f** Protein level of OMD in LPS-treated hDPSCs. The results are expressed as mean ± SD (*P < 0.05, **P < 0.01, and ***P < 0.001)

The human healthy and inflamed dental pulp specimens were collected, and the expression profile of OMD in dental pulpitis was explored using hematoxylin and eosin (HE) and immunofluorescence staining (Fig. 1a–c). The inflammatory infiltration area in inflamed pulp tissue sections was located through immunofluorescence staining of TNFα. Compared with the healthy individuals, the fluorescence intensity of TNFα was concentrated surrounding the vessels and expressed in the regional ECM. Abundant OMD expression was observed in the healthy sample sections, whereas few fluorescence signals of OMD were obtained in the inflammatory site of pulpitis tissues. These indicated decreased OMD expression in inflamed dental pulp tissues, suggesting the involvement of OMD in pulpitis progression. LPS was introduced to stimulate hDPSCs to mimic the inflammatory injury of pulpitis in vitro. First, hDPSCs were exposed to different concentrations (0.1, 1.0, and 10 μg/mL) of LPS separately for 1, 3, and 5 days. The CCK8 results displayed adequate cell viability in all groups throughout the LPS cultivation period (Fig. 1d). Only a slight proliferation trend of hDPSCs was detected on day 3, indicating little effect of LPS on cell proliferation. These suggested no cytotoxicity of LPS at concentrations ≤10 µg/mL within 5 days. The cells were exposed to LPS for 24 h to investigate the alteration of OMD levels during inflammatory response in cultivated hDPSCs. The mRNA and protein expression levels of OMD in hDPSCs decreased with the progressive increase in LPS concentration (Fig. 1e and f). The quantification revealed that the expression of OMD was obviously downregulated, especially with 10 µg/mL LPS application. Together, these results unveiled a significant correlation between OMD and LPS-induced inflammatory responses in hDPSCs.

### OMD-suppressed proinflammatory factor production and NLRP3 inflammasome activation in LPS-induced hDPSCs

**Fig. 2:**
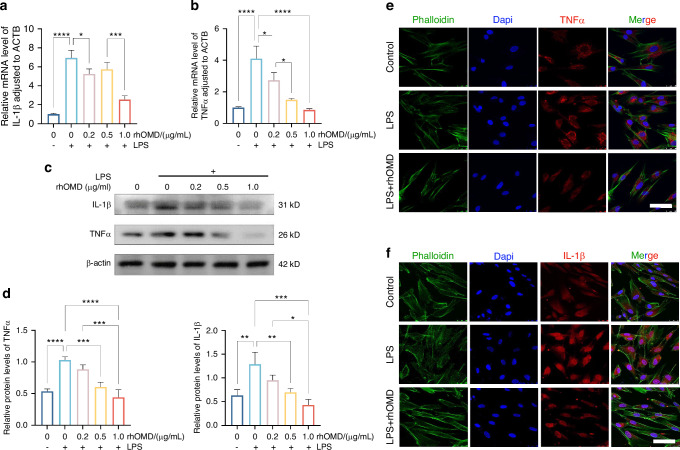
OMD suppressed the proinflammatory factor production of hDPSCs induced by LPS. **a** and **b** RT-qPCR analysis measuring the changes in mRNA expression of *IL-1β* and *TNFα* in hDPSCs cultured with 1 μg/mL of LPS and 0–1 μg/mL of rhOMD for 24 h. **c** WB assay showing the changes in protein levels of IL-1β and TNFα. **d** Quantitative analysis of the protein expression in (**c**). **e** and **f** Visualization of the expression of IL-1β and TNFα in hDPSCs treated with 1 μg/mL of LPS alone or combined application of 1 μg/mL of LPS and 1 μg/mL of rhOMD using cell immunofluorescence assay (scale bar: 50 μm). The results are expressed as mean ± SD (*P < 0.05, **P < 0.01, ***P < 0.001, and ****P < 0.000 1)

**Fig. 3:**
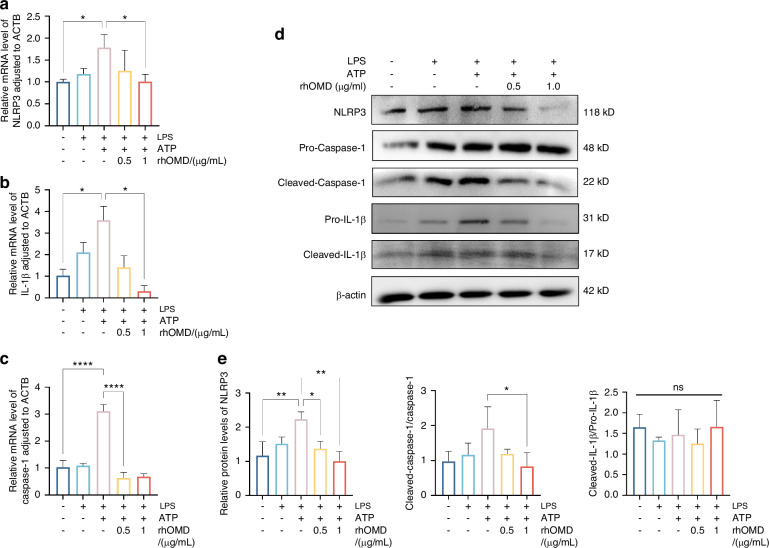
OMD suppressed the NLRP3 inflammasome activation of hDPSCs induced by LPS/ATP. **a**–**c** RT-qPCR assay showing the changes in the levels of NLRP3 inflammasome-related factors such as *NLRP3*, *IL-1β*, and *Caspase-1* in hDPSCs stimulated by combined administration of 1 µg/mL of LPS and 0.5–1.0 µg/mL of rhOMD for 6 h followed by the treatment with 1 mM ATP for another 30 min. **d** WB assay results of NLRP3, pro-caspase-1, cleaved-caspase-1, pro-IL-1β, and cleaved-IL-1β. **e** Quantitative analysis of the protein expression in (**d**). The results are expressed as mean ± SD (*P < 0.05, **P < 0.01, and ^****^P < 0.000 1; ns means no significance)

Initially, CCK8 results suggested that the combined application of LPS (0.1, 1, and 10 µg/mL) and rhOMD (0.5, 1, and 2 µg/mL) to hDPSCs within 2 days had no toxicity on cells (Supplementary Fig. 1). The production of inflammatory factors, including IL-1β and TNFα, and NLRP3 inflammasome-associated molecules, was measured to elucidate the potential effect of OMD in LPS-induced inflammatory responses. RT-qPCR, Western blotting (WB), and cell immunofluorescence were employed after treating the hDPSCs with LPS (an equal concentration of 1 µg/mL) and rhOMD (a concentration of 0.2, 0.5, or 1 µg/mL) for 24 h. The RT-qPCR results indicated that the production of proinflammatory factors by cells exposed to 1 µg/mL LPS for 24 h obviously increased 6.9-fold of *IL-1β* and 4.1-fold of *TNFα* compared with the control group. In contrast, the *IL-1β* and *TNFα* expression levels notably decreased in a concentration-dependent manner in the rhOMD application group (Fig. 2a and b). Correspondingly, the protein levels of IL-1β and TNFα significantly decreased under the application of 0.5 and 1.0 µg/mL rhOMD (Fig. 2c and d). The inhibited expression of IL-1β and TNFα in the LPS + rhOMD group (1 µg/mL) was further visualized using cell immunofluorescence staining (Fig. 2e and f, Supplementary Fig. 2). These observations supported the protective role of OMD in suppressing the proinflammatory factors against LPS-stimulated inflammation. Furthermore, NLRP3 inflammasome is a critical regulator for immune reactions during pulpitis progression, which activates caspase-1 into cleaved-caspase-1. The maturation of pro-IL-1β into cleaved-IL-1β was achieved by the activated caspase-1.^27–29^ The mRNA expression of inflammasome activation–associated molecules was decreased after combined administration of 1 µg/mL LPS and 0.5–1.0 µg/mL rhOMD for 6 h followed by treatment with 1 mmol/L adenosine triphosphate (ATP) for another 30 min (Fig. 3a–c). The experimental findings showed that 1 µg/mL rhOMD could effectively mitigate the protein expression of NLRP3 and attenuate the cleavage of pro-caspase-1 compared with that in the LPS/ATP-stimulated group (Fig. 3d and e). The protein expression of pro-IL-1β and cleaved-IL-1β was significantly suppressed under the application of rhOMD. However, the ratio of cleaved-IL-1β/pro-IL-1β showed no significant difference after applying rhOMD (Supplementary Fig. 3). Collectively, upon LPS or LPS/ATP stimulation, OMD intended to inhibit the excessive formation of proinflammatory cytokines and the activation of NLRP3 inflammasome, which were identified as crucial immunoregulators during the disease pathogenesis.^30^

### OMD inactivation promoted the inflammatory injury in vitro and in vivo

**Fig. 4:**
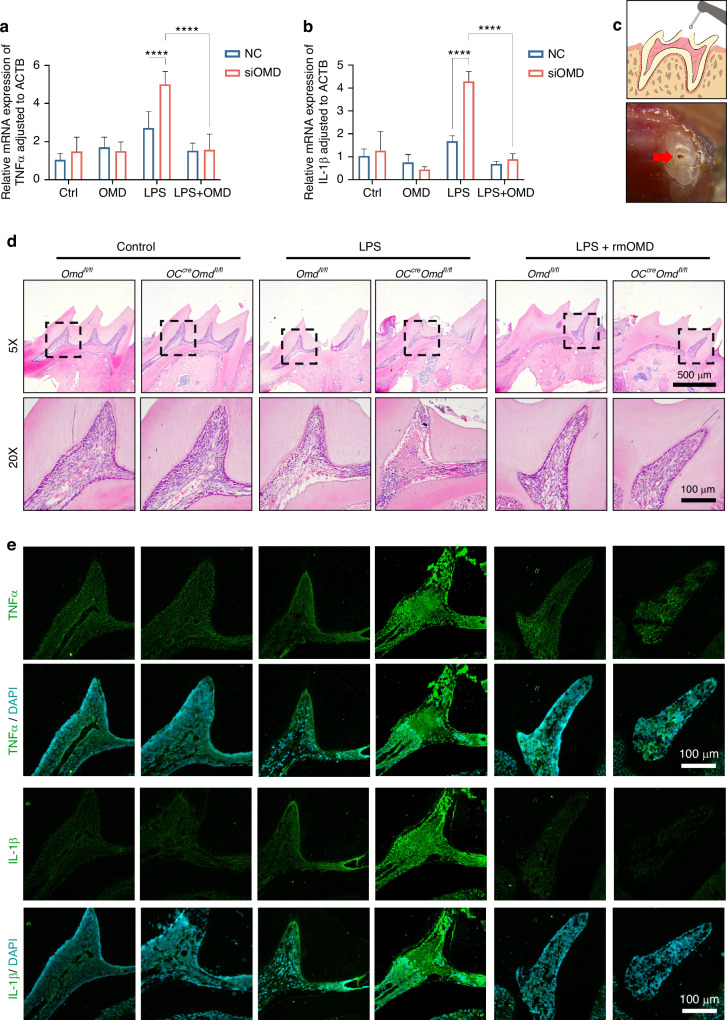
OMD inactivation accelerated the inflammatory response in LPS-induced hDPSCs and LPS-treated mice pulp tissues. **a**, **b** The mRNA expression of *TNFα* and *IL-1β* in siOMD hDPSCs treated with 1 μg/mL of rhOMD alone, 1 μg/mL of LPS alone, or combined 1 μg/mL of LPS and 1 μg/mL of rhOMD for 24 h. **c** Graphical illustration of the construction of the pulpal inflammatory mouse model and a representative occlusal view of the surgical processes, with a red arrow indicating the exposed pulp chamber treated with either LPS alone or a combination of LPS and rmOMD then sealed. **d**, **e** HE and immunofluorescence staining of TNFα and IL-1β, with ×5 magnification (scale bar: 500 μm) and ×20magnification (scale bar: 100 μm). Results are expressed as mean ± SD (****P < 0.000 1)

OMD inactivation was established to ascertain the protective role and regulatory mechanism of OMD in pulpal inflammation. In vitro, OMD was silenced via siRNA transfection in hDPSCs. The knockdown efficiency of OMD was first validated to select the optimized siRNA (Supplementary Fig. 4). The results indicated that *OMD* knockdown induced no significant alterations in the production of *IL-1β* and *TNFα* under regular cultivation. However, under LPS stimulation (1 µg/mL for 24 h), *OMD* knockdown significantly increased the expression of *IL-1β* by 2.5-fold and of *TNFα* by 2.4-fold (Fig. 4a and b). This increasing trend generated by *OMD* knockdown could be attenuated under the rhOMD administration. OSTEOCALCIN-Cre (OC-Cre) is extensively employed to target mesenchymal stromal cells, osteoblasts, and odontoblasts. In vivo, an experimental pulpal inflammatory model was introduced in conditional *Omd* knockout mice. *Omd* mRNA expression of the maxilla-containing molars significantly decreased in the mutant mice compared with the wild-type mice (Supplementary Fig. 5a). The OMD level in pulp tissue was detected using HE and immunofluorescence staining. As shown in Supplementary Fig. 5b, high OMD expression was observed near the dentin structure, whereas a significant reduction in OMD expression was detected in *OC-Cre*; *Omd*^fl/fl^ mice. Subsequently, surgery was conducted on the mice to detect the alteration of the inflammatory environment under OMD inactivation (Fig. 4c). The maxillary first molars were harvested following 24-h treatment with LPS in the pulpal chamber to induce inflammation. The inflamed pulp tissue adjacent to the exposed region displayed localized necrosis and impaired odontoblast cell layer (Fig. 4d). The mean fluorescence intensity of proinflammatory factors in the pulp tissue was used to determine the inflammatory degree in response to LPS administration. The results showed that the expression of IL-1β and TNFα significantly increased in the pulp tissue of *OC-Cre*; *Omd*^fl/fl^ mice compared with the *Omd*^fl/fl^ mice, whereas the inflammatory degree subsided under the rmOMD application (Fig. 4e, Supplementary Fig. 6). Higher expression of proinflammatory factors was distributed in the area adjacent to the dentin and the coronal cavity. Compared with the *Omd*^fl/fl^ mice, the coronal pulp tissue of *OC-Cre*; *Omd*^fl/fl^ mice exhibited necrotic alteration and a disordered odontoblast layer. The inflamed area even infiltrated part of the root pulpal tissue. Collectively, OMD inactivation might be capable of interfering with the pulpal inflammatory environment, supporting OMD as a regulatory factor during immune response in the pulp tissue.

### IL1R1/NF-κB signaling pathway might be involved in OMD-induced regulation

**Fig. 5:**
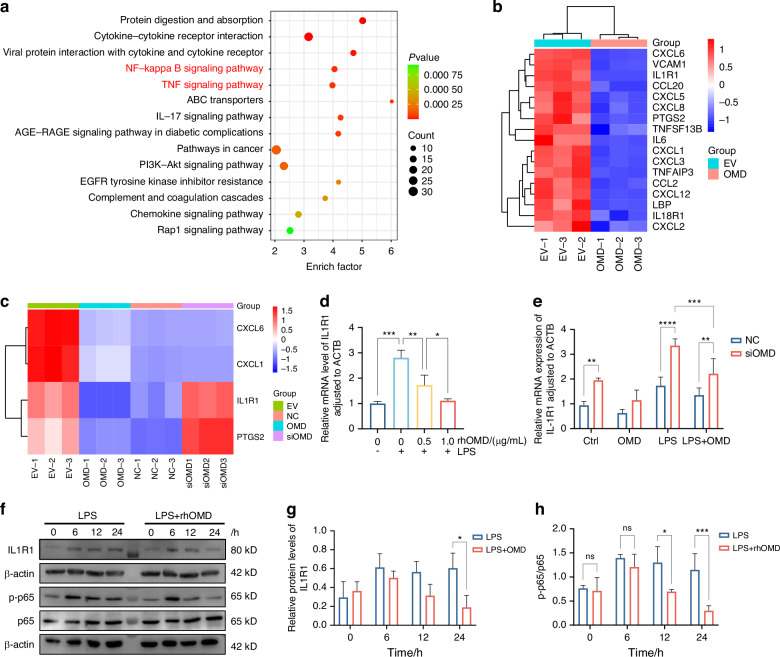
IL1R1/NF-κB signaling pathway might be involved in the OMD-modulated inflammation process. **a** KEGG enrichment analysis revealing the significant enrichment of NF-κB and TNF signaling pathways in OMD-overexpressing hDPSCs. **b** Cluster expression heat map showing the changes in key genes related to NF-κB and TNF signaling pathways. **c** Cluster heatmap showing the expression of co-occurring differentially expressed genes related to immune response. **d** RT-qPCR showing the expression alteration of IL1R1 in hDPSCs after the application of rhOMD (0.5 and 1 µg/mL) for 24 h. **e** RT-qPCR results demonstrating the inhibitory effect of OMD on the *IL1R1* expression in NC and siOMD hDPSCs. **f** WB showing the protein level of IL1R1 and the ratio of p-p65/p65 in hDPSCs induced by 1 μg/mL of LPS alone or combined 1 μg/mL of LPS and 1 μg/mL of rhOMD for 0, 6, 12, or 24 h. **g** and **h** Quantitative analysis of the protein expression in (**f**). The results are expressed as mean ± SD (ns means no significance; *P < 0.05, **P < 0.01^,^ and ***P < 0.001)

The RNA sequencing data from a previous study, in which OMD-overexpressing hDPSCs were constructed through lentiviral transfection, were analyzed to further explore the underlying mechanism of the OMD effect in hDPSCs.^22^ A total of 478 differentially expressed genes (|fold change| ≥ 2, *P* < 0.01) were detected in the OMD-overexpression group compared with the control group with empty vectors. Among these, 99 genes were upregulated, whereas 379 were downregulated. The Kyoto Encyclopedia of Genes and Genomes (KEGG) enrichment analysis showed that NF-κB and TNF signaling pathways were significantly enriched, which were both crucial pathways regulating the inflammatory response (Fig. 5a). As shown in Fig. 5b, 17 genes involved in NF-κB and TNF signaling pathways were found to be significantly downregulated after OMD overexpression. Moreover, gene set enrichment analysis (GSEA) was introduced to focus on gene sets. The results showed related key genes of NF-κB (NES: –1.94, *P* < 0.001, FDR: 0.001) and TNF (NES: –1.68, *P* < 0.001, FDR: 0.046) signaling pathways, supporting that OMD overexpression might impact these two pathways (Supplementary Fig. 7a and b). Consistently, the presence of OMD significantly downregulated the phosphorylation of p65 after 12 and 24 h, supporting the inhibitory effect of OMD on the NF-κB signaling pathway (Fig. 5f). Furthermore, the transcriptome analysis of OMD-knockdown hDPSCs was performed to identify the key genes involved in OMD regulation. The Venn diagram visualized the number of co-occurring differentially expressed genes in the OMD-overexpressing hDPSCs and OMD-knockdown hDPSCs (Supplementary Fig. 7c). Among the 81 overlapping genes, the expression of inflammation-related genes regulated by OMD was visualized using a heatmap (Fig. 5c). Within this gene cluster, *CXCL1* and *CXCL6* were identified as key players in cell recruitment during the immune response. Additionally, *PTGS2* was regulated by proinflammatory cytokines and involved in initiating inflammation. *IL1R1* served as an important molecule to activate the NF-κB pathway. As expected, the mRNA expression of *IL1R1* after 24 h showed a decreasing trend with increased rhOMD concentration (Fig. 5d). In contrast, OMD knockdown in hDPSCs increased the *IL1R1* expression upon either regular cultivation or LPS stimulation (Fig. 5e). OMD administration for 24 h obviously decreased the protein level of IL1R1 to approximately 32% compared with that in the LPS-treated group (Fig. 5f). The findings confirmed that the regulatory effects of OMD decreased *IL1R1* expression.

### OMD interacted with IL1R1 to regulate inflammatory reactions

**Fig. 6:**
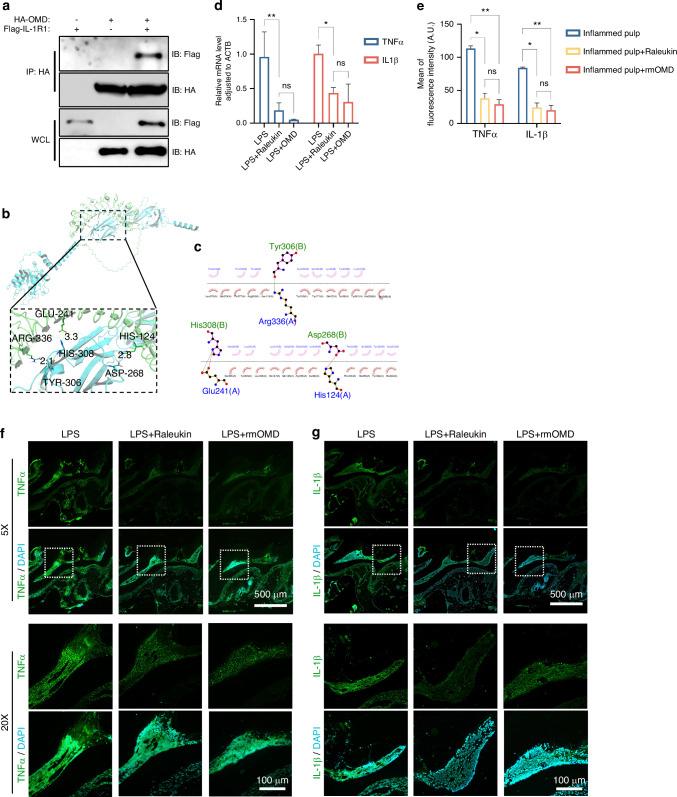
Blockage of IL1R1 attenuated the increased inflammation generated by OMD deficiency. **a** IP assays were performed in HEK293T cells transfected with HA-OMD and Flag-IL1R1. IB analysis with indicated antibodies was shown. **b** Three-dimensional illustration of the interaction between OMD (the green chain) and IL1R1 (the blue chain), with the yellow dotted lines representing the hydrogen bond interaction. **c** Two-dimensional illustration displaying detailed amino acids involved in the interaction. The amino acids in the OMD protein chain are marked in green, whereas those in the IL1R1 chain are marked in blue. **d** mRNA expression of *TNFα* and *IL-1β* in siOMD hDPSCs induced by 1 μg/mL of LPS alone, 1 μg/mL of LPS and 1 μg/mL of rhOMD combined, or 1 μg/mL of LPS and 1 μg/mL of Raleukin combined. **e**–**g** Immunofluorescence staining and quantitative results showing TNFα and IL-1β expression in the pulp tissues of *OC*-Cre; *Omd*^fl/fl^ mice treated with 1 mg/kg of LPS alone, 1 mg/kg of LPS and 1 mg/kg of rmOMD combined, or 1 mg/kg of LPS and 1 mg/kg of Raleukin combined. Representative images in (**f**) and (**g**) were obtained under ×5 magnification (scale bar: 500 μm) and ×20 magnification (scale bar: 100 μm). Results are expressed as mean ± SD (ns, no significance; *P < 0.05, **P < 0.01, ***P < 0.001, and ****P < 0.000 1)

**Fig. 7:**
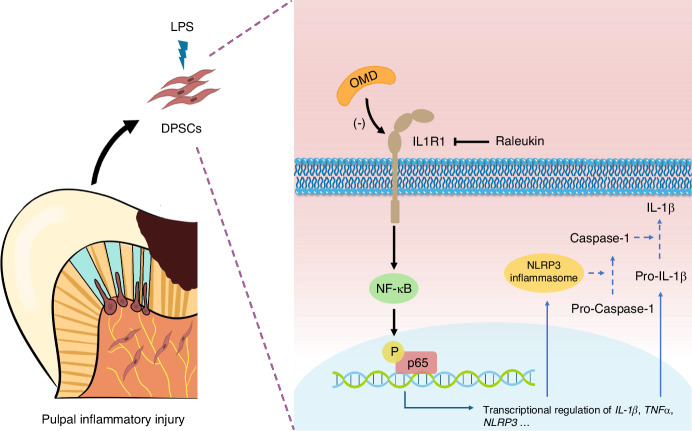
Schematic illustration of the OMD-induced regulation in hDPSCs upon LPS stimulation. OMD suppressed the LPS-induced inflammatory process, decreasing the secretion of proinflammatory factors and the activation of NLRP3 inflammasome. OMD may interact with the membrane receptor of IL1R1 and inhibit the NF-κB signaling pathway to exert a protective role during pulpal inflammation

The mechanism underlying the OMD-induced downregulation of IL1R1 was further explored. The Co-IP analysis revealed a specific interaction between OMD and IL1R1 in HEK293T cells ectopically overexpressing OMD and IL1R1 (Fig. 6a). Furthermore, the molecular docking analysis was conducted for predicting the residues involved in the binding of OMD and IL1R1. A total of 100 docking modes were constructed, and the top 10 of these were scored. The mode with the highest docking score (−733.73 kcal/mol) was selected following the exclusion of the binding site located at the intracellular segment of IL1R1. As demonstrated in Fig. 6b and c, the interaction between OMD and the extracellular segment of IL1R1 was noted. The interaction types included hydrogen bonds and hydrophobic interaction. Multiple amino acids were involved in the binding. The hydrogen bond consisted of the NH atom of ARG-336 from OMD protein and the O atom of TYR-306 from IL1R1 protein (with a distance of 2.1 Å); the O atom of GLU-241 from OMD and the NH atom of HIS-308 from IL1R1 (with a distance of 3.3 Å); and the NH atom of HIS-124 from OMD and the O atom of ASP-268 from IL1R1 (with a distance of 2.8 Å). These results indicated that OMD might closely interact with IL1R1 and inhibit the downstream signaling pathway. Subsequently, the alteration upon IL1R1 blockage was investigated. Raleukin, the IL1R antagonist, was introduced in vitro and in vivo. We assessed the inflammatory alterations in the presence of Raleukin under the OMD-knockdown pattern (Fig. 6d). The *IL-1β* and *TNFα* levels subsided significantly with IL1R1 blockage in siOMD-transfected hDPSCs compared with the LPS group, indicating that the blockage of IL1R1 could rescue the accelerated inflammation caused by OMD deficiency. Moreover, no significant difference was found between the effect of rhOMD or Raleukin on the diminished production of IL-1β and TNFα in LPS-induced siOMD hDPSCs. Furthermore, the inflammatory alteration of IL1R1 blockage was estimated in vivo using the pulpitis model in *OC-Cre*; *Omd*^fl/fl^ mice (Fig. 6e–g). Consistently, the immunofluorescence results demonstrated that both IL1R1 blockage and rmOMD compensation could alleviate the excessive inflammation induced by OMD deficiency. These results suggested that OMD deficiency could disturb and exacerbate the LPS-induced inflammatory pulpal environment, whereas OMD might regulate the inflammatory process by interacting with IL1R1 (Fig. 7).

## Discussion

The prerequisite for repairing the injured pulpal tissues is an initial inflammation response. Despite the known importance of the immunoregulatory functions of hDPSCs in the repair of pulp tissues, the underlying mechanisms to orchestrate moderate inflammatory processes remain unclear.^6,31^ The regenerative endodontics procedure emphasizes the dynamic orchestration of the interactions between stem cells, bioactive molecules (BMs), and ECM.^32^ As such, a promising BM capable of regulating various cellular functions was significantly valued to optimize the repair of pulpal inflammatory injury. The regulatory mechanisms between BM and the target stem cells need to be explored.

Several studies have focused on the effects of SLPRs on the immune response. For example, biglycan and decorin, the two best-studied class I SLRP members, have been proven to interact with Toll-like receptors (TLR) 2 and 4, leading to the initiation of the inflammatory response.^33,34^ Similarly, Lumican displayed an upregulation in osteoarthritis and participated in the TLR-4-initiated proinflammatory events.^35^ OMD is a keratan sulfate SLRP expressed in bone and teeth. Our previous studies demonstrated the role of OMD as a bioactive regulatory molecule promoting osteogenesis and inhibiting apoptosis in hDPSCs.^21,22^ Increasing evidence has also shown the involvement of OMD in some inflammatory environments.^36^ For example, lower levels of OMD were detected in the serum of patients with OA, and its gene expression was downregulated in OA labrum–derived fibrochondrocytes.^25,37^ This reduction in OMD levels was associated with the proinflammatory environment in degenerated OA labrum, where excessive IL-1β levels were identified as a key factor in the downregulation of OMD. Mechanistically, the complement system plays a crucial role in inflammatory joint diseases. Studies have shown that OMD promotes complement activation by binding to C1q. However, this activation is simultaneously regulated by the binding of OMD to the complement inhibitor C4b-binding protein, which helps prevent excessive complement activation. Hence, OMD may play a regulatory role in inflammatory responses, suggesting its potential as a therapeutic target for treating inflammatory diseases.^26,38^ This study was novel in revealing the involvement of OMD in pulpal inflammation. The OMD expression was found to be decreased in the inflamed pulp specimens and LPS-induced hDPSCs. Furthermore, OMD probably played a protective role in LPS-induced inflammation in hDPSCs. The rhOMD administration significantly attenuated the LPS-induced production of IL-1β and TNFα in hDPSCs. Both IL-1β and TNFα are typical proinflammatory cytokines participating in the pathological process of pulpitis. These cytokines are also recognized as targets for developing anti-cytokine therapies for pulpitis management.^39^ Moreover, SLRP family members exerted different functions in the ECM by binding to the target receptors or other factors upon inflammatory stimulation. Considering that the members of the SLRP family share a similar protein structure, it is reasonable that SLRP networks in ECM may be complicated to cluster different receptors and fine-tune the downstream signaling pathways, which need further exploration.^40^

Based on the aforementioned result that OMD inhibited the secretion of IL-1β, the underlying mechanism was explored. Briefly, the formation of pro-IL-1β was induced by the NF-κB signaling pathway, while the maturation of pro-IL-1β into cleaved-IL-1β was achieved by the activated caspase-1, which depended on the NLRP3 inflammasome.^41^ Studies demonstrated that some SLRPs, such as biglycan, activated the formation of NLRP3 inflammasome.^42^ This study demonstrated a suppressive effect of OMD administration on the expression of NLRP3 and the activation of caspase-1. Despite the decreased activation of caspase-1, OMD could decrease the expression of pro-IL-1β and cleaved-IL-1β simultaneously, implying that OMD might regulate both the synthesis and maturation of IL-1β. Meanwhile, IL-1β was also a proinflammatory factor commonly detected in the periapical periodontitis tissues.^43^ Based on the aforementioned findings, the role and potential application of OMD in managing periapical periodontitis can be explored in future studies.

We further investigated the alterations upon OMD inactivation to ascertain the effect of OMD on inflammatory response. In vitro, the *OMD* knockdown significantly increased the production of *IL-1β* and *TNFα* under LPS stimulation, whereas no alteration was found under regular cultivation. In vivo, multiple studies have successfully established a mouse model of pulpitis. One example is pulpitis induced by exposing the pulp tissues of a mouse molar to the oral environment, which is similar to the clinical situation of a teeth fracture with pulp exposure. The inflammatory injury to pulp tissue gradually worsened with the prolongation of pulp exposure time. By 12 h, the inflammatory infiltrate was limited to the coronal pulp. After 24 h, disorganization of the odontoblastic layer, inflammatory cell infiltrates, and localized pulp necrosis were observed. However, after 48 h of pulpal exposure, the odontoblastic lining was completely destroyed and extensive pulp necrosis was evident in the coronal pulp.^44^ The levels of inflammatory markers were significantly correlated with the severity of inflammatory injury. The expression of IL-1β and TNFα continued to increase in the first 24 h, with IL-1β expression peaking after 12 h and TNF-α after 48 h.^45^ In this study, we employed an alternative method to construct the animal model so as to avoid the uncontrolled impact of the oral bacteria: 1 mg/kg LPS dissolved in phosphate-buffered saline (PBS) was injected into the mechanically exposed pulpal cavity to induce controlled pulpal inflammation.^46^ Previous studies showed that osteocalcin and proinflammatory factors were increasingly produced and primarily distributed adjacent to the areas of inflammation and reparative dentine sites in response to the pulpal injury in an experimental pulpitis rat model.^47^ In this study, the expression of osteocalcin (OCN) and TNFα in a mouse model of pulpitis was investigated using immunofluorescence staining. The results demonstrated a significant increase in OCN and TNF-α expression in the inflamed pulp tissue compared with the normal pulp tissue. Moreover, OCN and TNFα were observed near the dentin structure and inflamed pulp tissue adjacent to the exposed region (Supplementary Fig. 8). This suggested that OCN might play a role in the reparative process in response to dental pulp inflammatory injury.^48^ Hence, *OC-Cre*; *Omd*^*flox/flox*^ mice were generated and used for constructing the mouse pulpitis model to investigate pulpal tissue repair, emphasizing the inflammatory alteration upon OMD deficiency. After 24 h of the surgery, the maxillary first molars of mice were collected to determine the degree of inflammation. The results showed that OMD deficiency increased LPS-induced pulpal inflammation. A severe phenotype of pulpal inflammation was observed in the *OC-Cre*; *Omd*^*flox/flox*^ mice, involving the existence of coronal pulpal necrosis and significantly increasing the expression of proinflammatory cytokines. A higher distribution of IL-1β and TNFα was detected adjacent to the hard dental structure, where the reparative processes were activated in response to the LPS stimulation.

The RNA sequencing of OMD-overexpressed hDPSCs was analyzed to further understand the regulatory mechanism of OMD during the inflammation of DPSCs. The NF-κB and TNF signaling pathways were significantly enriched. Owing to the cytosolic domain of significant homology, IL1R1, together with TLR, belonged to the same IL-1R/TLR receptor superfamily and activated similar downstream including the NF-κB signaling pathway.^49,50^ In this study, enriched reduction of IL1R1 expression was discovered in the OMD-overexpressed hDPSCs. OMD treatment in hDPSCs could restrain IL1R1 expression and the phosphorylation of p65 extremely after 24 h. As such, we speculated that the IL1R1/NF-κB signaling pathway might be involved in the OMD regulation of the inflammatory process. Subsequently, the interaction between OMD and IL1R1 was successfully verified by Co-IP analysis and molecular docking analysis to further discover the molecular mechanism. In vivo, the blockage of IL1R1 through raleukin could rescue the excessive inflammation generated by OMD deficiency, suggesting the involvement of IL1R1 in the OMD-induced regulatory process and the interaction between OMD and IL1R1. Additionally, the effects of rmOMD and Raleukin on the rescue of excessive inflammation in *OC-Cre; Omd*^*flox/flox*^ mice seemed to be similar. IL1R-induced regulation was confirmed to potentiate the RANKL-mediated osteoclastogenesis, whereas OMD bound to RANKL and inhibited the osteoclast activity.^51^ This suggested that OMD might orchestrate multifunction in the biological events of hDPSCs.

Collectively, this study suggested a novel role of OMD as an inhibitory regulator during pulpal inflammation. It also verified the involvement of the NF-κB signaling pathway and its upstream receptor IL1R1 in the OMD-induced regulation. The application of OMD decreased IL1R1 expression. Besides, an interaction might exist between OMD and IL1R1 to result in the suppression of proinflammatory productions. Therefore, the understanding of versatile functions induced by OMD in hDPSCs was advanced. Besides promoting osteogenesis and inhibiting apoptosis investigated, as demonstrated in our previous study, OMD also optimized the immunoregulatory function of hDPSCs. All these promising bioactivities supported the potential application of OMD and hDPSCs in the regenerative endodontics procedure.

## Material and methods

### HE and immunofluorescence staining

This study was approved by the ethics committee of the Shanghai Ninth People’s Hospital affiliated with the Shanghai Jiao Tong University, School of Medicine, China (Document No. SH9H-2024-T414-1). Healthy pulp tissues were collected from the third molars of patients, which were extracted for orthodontic treatment. Inflamed pulp tissues were obtained from patients undergoing clinical assessment and diagnosed with irreversible dental pulpitis by endodontic specialists. The specimens were fixed with 4% paraformaldehyde (PFA; Sangon Biotech, Shanghai, China), followed by paraffin embedding and sectioning at 5-μm thickness. HE staining was performed using an HE staining kit (Solarbio, Beijing, China). For immunofluorescence staining, the deparaffinized sections were subjected to antigen retrieval using the improved citrate antigen retrieval solution (Beyotime, Shanghai, China) and blocked with 5% serum for 1 h. Then, the specimens were incubated with primary antibodies overnight at 4 °C and then with secondary antibodies for 1 h at room temperature. The primary antibodies used in this study are listed in Supplementary Table 1. The secondary antibodies used were Alexa Flour 488-conjugated anti-rabbit antibody (1:1 000; Cell Signaling Technology Inc., MA, USA). Finally, the sections were covered with an antifade mounting medium with DAPI (Beyotime). The images were captured using a fluorescence microscope equipped with a digital camera (Leica Microsystems, Wetzlar, Germany).

### Cell culture

Healthy and intact human third molars were collected from individuals aged 18–22 years at the Ninth People’s Hospital affiliated with Shanghai Jiao Tong University, School of Medicine. Primary hDPSCs were isolated and cultured, and their multilineage differentiation capability was identified as described in previous studies.^20^ In brief, hDPSCs were cultured in a complete medium consisting of high-glucose Dulbecco’s modified Eagle medium (Gibco-BRL, NY, USA) supplemented with 10% fetal bovine serum (Gibco-BRL) and 1% penicillin/streptomycin (Gibco-BRL) at 37 °C in the presence of 5% CO_2_. All subsequent experiments were conducted using hDPSCs from the second through fifth passages. The hDPSCs were incubated with LPS derived from *Escherichia coli* O111:B4 (S1732; Beyotime) to induce an inflammatory response.^52^ The hDPSCs were co-cultured with LPS and rhOMD (Sino Biological Inc., Shanghai, China) to investigate the potential effect of OMD against LPS-induced inflammation. Additionally, the NLRP3 activator of adenosine triphosphate (ATP; Cayman Chemical, MI, USA) was used to assess the effect of OMD during NLRP3 inflammasome activation.

### Cell viability assay

The cell viability of hDPSCs was examined using the cell counting kit-8 (CCK8; Dojindo Laboratories, Kumamoto, Japan). HDPSCs were seeded into 96-well plates at an initial density of 2 000 cells per well and stimulated with LPS (0.1, 1.0, and 10 μg/mL separately). The medium was replaced with a complete culture medium containing 10% CCK8 buffer after 1, 3, and 5 days of cultivation, and the cells were incubated for 2 h. The absorbance at 450 nm was measured using a microplate reader (Bio-Tek, VT, USA).

### Reverse transcriptase-quantitative polymerase chain reaction

Total RNA of hDPSCs was isolated using TRIzol reagent (Invitrogen, Carlsbad, CA, USA), and the extracted RNA was quantified with a NanoDrop spectrophotometer (Thermo Scientific, NC, USA). A PrimeScript RT reagent kit (Takara, Kusatsu, Japan) was used for the reverse transcription of 1 000 ng RNA. The gene transcription levels were quantified using TB Green Premix Ex Taq (Takara) on a LightCycler 480 II instrument (Roche, Basel, Switzerland). The mRNA expression was normalized to β-actin and calculated using the 2^−∆∆Ct^ method. The primer sequences are listed in Supplementary Table 2.

### Western blotting

HDPSCs were lysed with a cell lysis buffer for WB (Beyotime), supplemented with protease and phosphatase inhibitor cocktail (Beyotime). The protein concentration was measured using the bicinchoninic acid (BCA) protein assay (Beyotime). Then, the cell lysates were combined with 5× SDS sample buffer and heated for 5 min. Further, 30 μg of protein was subjected to 10% SDS polyacrylamide gel electrophoresis and transferred onto an active 0.2 μm polyvinylidene difluoride membrane (Millipore, CA, USA). The blots were blocked with 5% (*w*/*v*) dry milk for 1 h and then incubated with primary antibodies overnight at 4 °C. The primary antibodies are listed in Supplementary Table 1. Subsequently, the blots were incubated with 1:5 000 anti-IgG-HRP (Santa Cruz Biotechnology, CA, USA) at room temperature for 1 h. The signals were obtained using WB Luminol Reagent (Santa Cruz Biotechnology). The WB experiments were repeated three times independently. The band intensity was quantified using ImageJ V1.8.0.

### Small interfering RNA transfection

OMD was silenced using small interfering RNA (siRNA) (HanBio Technology, Shanghai, China) to investigate the inflammatory pattern alteration under OMD deficiency. The hDPSCs were transfected with siRNA using Lipofectamine 3000 reagent (Lipo3000; Invitrogen). The transfection efficiency was determined using RT-qPCR after treating hDPSCs with the siRNA/Lipo 3 000 formulation for 48 h. The siRNA sequences are shown in Supplementary Table 3 and the negative control (NC)-hDPSCs were used as control.

### Processing of RNA sequencing data

The raw datasets for RNA sequencing of OMD overexpression in hDPSCs were processed as described previously.^22^ Additionally, the RNA sequencing analysis of OMD knockdown samples was also conducted to screen out the key genes during OMD regulation. The hDPSCs were transfected with siOMD for 48 h, and the NC-hDPSCs were used as control. The RNA samples were sent to Novogene Bioinformatics Institute, Beijing, China, for transcriptome analysis. The gene expression levels were estimated by fragments per kilobase of transcript per million fragments mapped. Differential expression analysis between the two groups was performed using DESeq2, which provides statistical routines for determining differential expression in digital gene expression data based on a negative binomial distribution model. The resulting *P* values were adjusted using Benjamini and Hochberg’s approach for controlling the false discovery rate.^53^ Genes with a |fold change| ≥ 2 and an adjusted *P*-value < 0.01 found by DESeq2 were assigned as differentially expressed. For the data displayed in Fig. 5a and b, the differentially expressed genes in OMD-overexpressing hDPSCs were processed for KEGG enrichment and cluster analyses using a free online platform (http://www.bioinformatics.com.cn/). For data shown in Supplementary Fig. 7a and b, GSEA was performed using the OECloud tools at http://cloud.oebiotech.com.

### Immunoblots (IB) and immunoprecipitation (IP)

The full-length human OMD and IL1R1 cDNA were inserted into pLEX-HA or pLEX-Flag vectors and verified by DNA sequencing to generate expression plasmids. HEK293T cells were transiently transfected with plasmids using Lipo 3000 (Invitrogen). Cells were lysed in EBC buffer (50 mmol/L Tris–HCl, pH 8.0, 120 mmol/L NaCl, 0.5% Nonidet P-40) supplemented with protease inhibitors (Beyotime), followed by pulse sonication for 20 s. The protein concentrations of lysates were measured using the BCA protein assay (Beyotime). Same amounts of whole-cell lysates (WCL) were used for IB. For IP, proper cell lysate was pre-cleared with protein A/G plus Sepharose (Santa Cruz) and then incubated with anti-Flag or anti-HA agarose beads for 2 h at 4 °C. The pellet of IP complexes was washed with NETN buffer (20 mmol/L Tris–HCl, pH 8.0, 100 mmol/L NaCl, 1 mmol/L EDTA, 0.5% Nonidet P-40) for 4 times, resolved by 1X loading buffer, and immunoblotted using the indicated antibodies. The details about primary antibodies are listed in Supplementary Table 1.

### Molecular docking analysis

Molecular docking analysis was employed to discover the interaction between OMD and IL1R1. The protein sequences of human OMD (UniProtKB Entry: Q99983) and human IL1R1 (UniProtKB Entry: P14778) were downloaded from UniprotKB. The crystal structures were predicted using the AlphaFold3 model at https://alphafoldserver.com/, which enabled protein modeling with improved accuracy for protein–protein interactions.^54^ Before molecular docking analysis, the protein structures underwent optimization and adjustment. *H*++3.0 (http://biophysics.cs.vt.edu/), a free, open-source web server, was employed for protonation under a constant pH value of 7.0.^55^ The UCSF Chimera software was then used to assign the charge under the Amber14SB force field. Subsequently, the HDOCK web server, available at http://dhock.phys.hust.edu.cn/, was used for protein–protein docking.^56^ The binding modes generated were set to 100, where the top 10 were scored using the knowledge-based scoring function ITScorePP. The binding mode of the max score was applied to PyMOL (version 2.5.7) for 3D mapping analysis and visualized using LigPlot (version 2.1).

### Animals

All animal procedures were approved by the Animal Care and Use Ethics Committee of the Shanghai Ninth People’s Hospital, Shanghai Jiao Tong University (Document No. SH9H-2024-A1354-1). OC-Cre is employed to target mesenchymal stromal cells, osteoblasts, and odontoblasts. The OC-Cre mice have been utilized in studies on bone-related inflammation, such as osteoarthritis.^57^ In this study, conditional *Omd* knockout mice were generated to investigate the influence of OMD inactivation on the immune response of dental pulp under inflammatory stimulation in vivo. *Omd*-floxed allele mice and *OC-Cre* mice were purchased from Cyagen Biosciences (Santa Clara, USA). The *Omd* gene was located on mouse chromosome 13, containing 4 exons, with the ATG start codon in exon 3 and the TAG stop codon in exon 4. The exon 3 was selected as the conditional knockout region. The *Omd* knockout mice were generated by breeding the floxed *Omd* mice (*Omd*^*flox/flox*^) with mice having two floxed *Omd* alleles and one allele of *OC*-Cre (*OC-Cre*; *Omd*^*flox/flox*^). All the mice were fed a regular chow and water ad libitum in an SPF environment.

### Construction of the pulpal inflammatory model

A mouse pulpal inflammatory model was constructed by operating 40 molars of 8-week-old male mice weighing about 20–25 g.^58,59^ The wild-type or mutant mice were randomly divided into four groups: control group (mice without treatment), inflamed pulp group, inflamed pulp + recombinant mouse OMD (rmOMD; Sino Biological Inc.) group, and inflamed pulp + Raleukin (Med Chem Express, NJ, USA; HY-108841) group. The suffering of animals was minimized. Briefly, the occlusal surfaces of the bilateral upper first molars of mice were drilled using a sterile *#*1/4 dental round bur under anesthesia until the pulp tissue was visible under a surgical microscope. Then, an endodontic K-file with a diameter of 0.15 mm was used to enlarge the coronal access to the pulp chamber. Subsequently, LPS (1 mg/kg), rmOMD (1 mg/kg), or Raleukin (1 mg/kg) dissolved in PBS solution was administered into the pulpal chamber, and then the cavity was sealed. Care was taken not to overfill the liquid before sealing the cavity, and the procedure was repeated three to four times. The mice were sacrificed 24 h after surgery. The maxillary bones were obtained and fixed in 4% PFA for 48 h. HE staining and immunofluorescence staining were conducted to assess the inflammatory degree alteration under OMD deficiency.

### Statistical analysis

The data were analyzed using GraphPad Prism 9.0 software (GraphPad, CA, USA) and expressed as mean ± standard deviation (SD) from at least three independent experiments. Significance was calculated using the Student *t*-test for pairwise comparisons and ordinary one-way analysis of variance (ANOVA) for multiple comparisons. Besides, two-way ANOVA followed by Tukey’s post hoc multiple comparison test was used to compare the NC and siOMD hDPSCs groups, as well as the *Omd*^*flox/flox*^ mice and *OC-*Cre; *Omd*^*flox/flox*^ mice groups. The statistical significance was set at *P < 0.05, **P < 0.01, ***P < 0.001, and ****P < 0.000 1.

## Availability of data and materials

The datasets used and/or analyzed in the current study are available from the corresponding author on reasonable request.

## Supplementary information

Supplementary Information

## Data Availability

The datasets used and/or analyzed in the current study are available from the corresponding author on reasonable request.
