# Supplementary material for: Osteomodulin modulates the inflammatory responses via the interleukin-1 receptor 1/nuclear factor-κB signaling pathway in dental pulpitis
Source: Int J Oral Sci. 2025 May 26;17:41. doi: 10.1038/s41368-025-00369-5 (PMC12104430; doi:10.1038/s41368-025-00369-5)
Supplement: Supplementary file 1 — Supplementary Information [file 41368_2025_369_MOESM1_ESM.docx]

**Supplementary Information**

**Supplemental Tables**

| **Supplementary Table 1. Primary antibodies for Western blotting and Immunofluorescence staining** | | |
| --- | --- | --- |
| Name | Application |  |
| OMD | WB, IF | Invitrogen, PA5-14382 |
| TNFα | WB, IF | Servicebio, GB11188-100 |
| IL-1β | WB, IF | ZEN BIO, 516288 |
| β-actin | WB | ZEN BIO, 380624 |
| NLRP3 | WB | Proteintech, 19771-1-AP |
| Caspase-1 | WB | Cell Signaling Technology, Human Reactive Inflammasome Antibody Sampler Kit II, #25620 |
| Cleaved-Caspase-1 | WB | Cell Signaling Technology, Human Reactive Inflammasome Antibody Sampler Kit II, #25620 |
| Pro-IL-1β | WB | Cell Signaling Technology, Human Reactive Inflammasome Antibody Sampler Kit II, #25620 |
| Cleaved-IL-1β | WB | Cell Signaling Technology, Human Reactive Inflammasome Antibody Sampler Kit II, #25620 |
| IL1R1 | WB | Abclonal, A5727 |
| Phospho-p65 | WB | Cell Signaling Technology, #3033 |
| p65  FLAG-tag rabbit  HA-tag rabbit  FLAG-tag mouse  HA-tag mouse | WB  Co-IP  Co-IP  Co-IP  Co-IP | ZEN BIO, 380172  Proteintech, 20543-1-AP  Sino Biological Inc, 100028-T36  Sigma-Aldrich, F3165  Santa Cruz, SC-7392 |

| **Supplementary Table 2. Primer sequences for RT-qPCR** | | |
| --- | --- | --- |
| Primers | Sequences |  |
| *OMD*-F  *OMD*-R | GGTGAACAACGAAGCACTAATGG  TGCTCCTTCTTGTTCTGGGC |  |
| *Omd*-F  *Omd*-R | GCACATTCAGCAACTCAACCTTCAG  CACATTCACCAGCCCGTCCATG |  |
| *β-actin*-F  *β-actin*-R | CCACGAAACTACCTTCAACTCCATC  AGTGATCTCCTTCTGCATCCTGTC |  |
| *IL-1β*-F  *IL-1β*-R | ATGGCTTATTACAGTGGCAATGAGG  AGTGGTGGTCGGAGATTCGTAG |  |
| *TNFα*-F  *TNFα*-R | CTCATCTACTCCCAGGTCCTCTTC  CGATGCGGCTGATGGTGTG |  |
| *NLRP3*-F  *NLRP3*-R | CTGTTCTATATCCACTGTCGG  CGGTCCTATGTGCTCGTCAA |  |
| *Caspase-1*-F  *Caspase-1*-R | CCTTAATATGCAAGACTCTCAAGGA  TAAGCTGGGTTGTCCTGCACT |  |
| *IL1R1*-F  *IL1R1*-R | AGTCCAGCGACGGTCACCTTC  ACGTGAGCCTCTCTTTGCAGTTTC |  |
| *GAPDH*-F  *GAPDH*-R | ACACCCACTCCTCCACCTTTG  TCCACCACCCTGTTGCTGTAG |  |

| **Supplementary Table 3. Sequences of siRNA** | |  |
| --- | --- | --- |
| Name | positive-sense strand | antisense strand |
| NC | UUCUCCGAACGUGUCACGUTT | ACGUGACACGUUCGGAGAATT |
| hs-OMD-si | GGUCAAACAAUACAACUAATT | UUAGUUGUAUUGUUUGACCTT |
| hs-GAPDH | GUGGAUAUUGUUGCCAUCATT | UGAUGGCAACAAUAUCCACTT |

**Supplemental Figures**

**
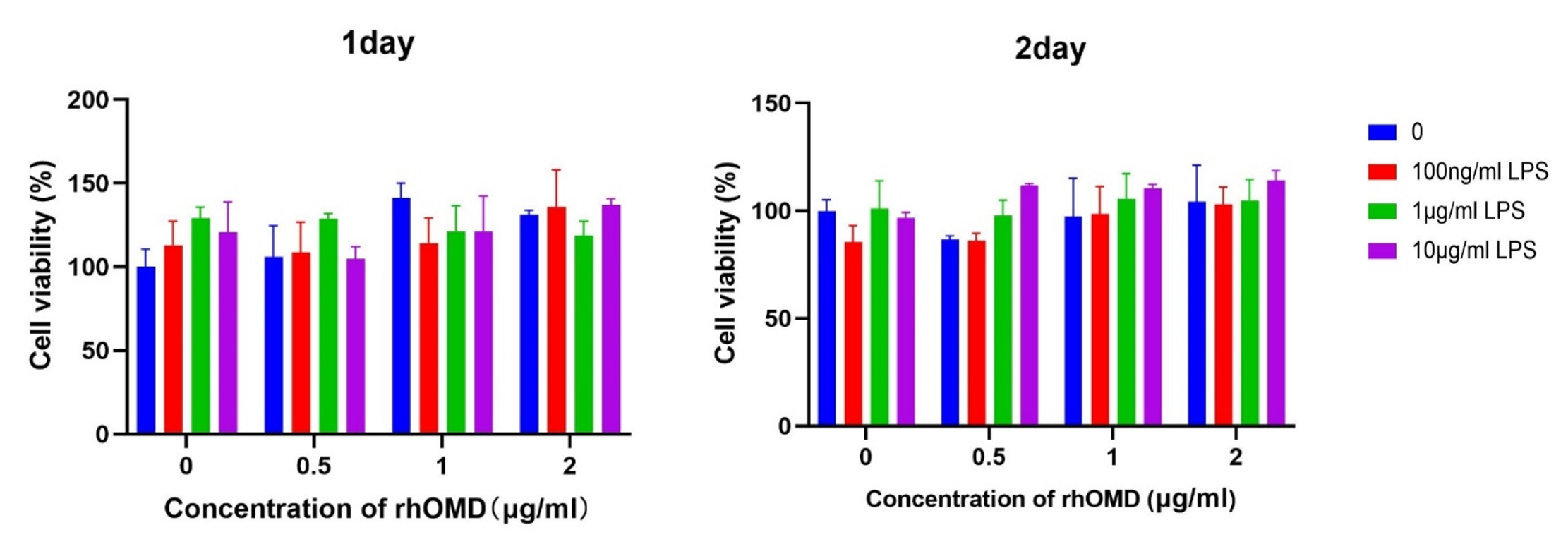
**

**Supplementary Figure 1.** The CCK8 results of the combined application of LPS (0, 0.1, 1, and 10µg/mL) and rhOMD (0, 0.5, 1, and 2 µg/ml) to hDPSCs within 2 days.

**
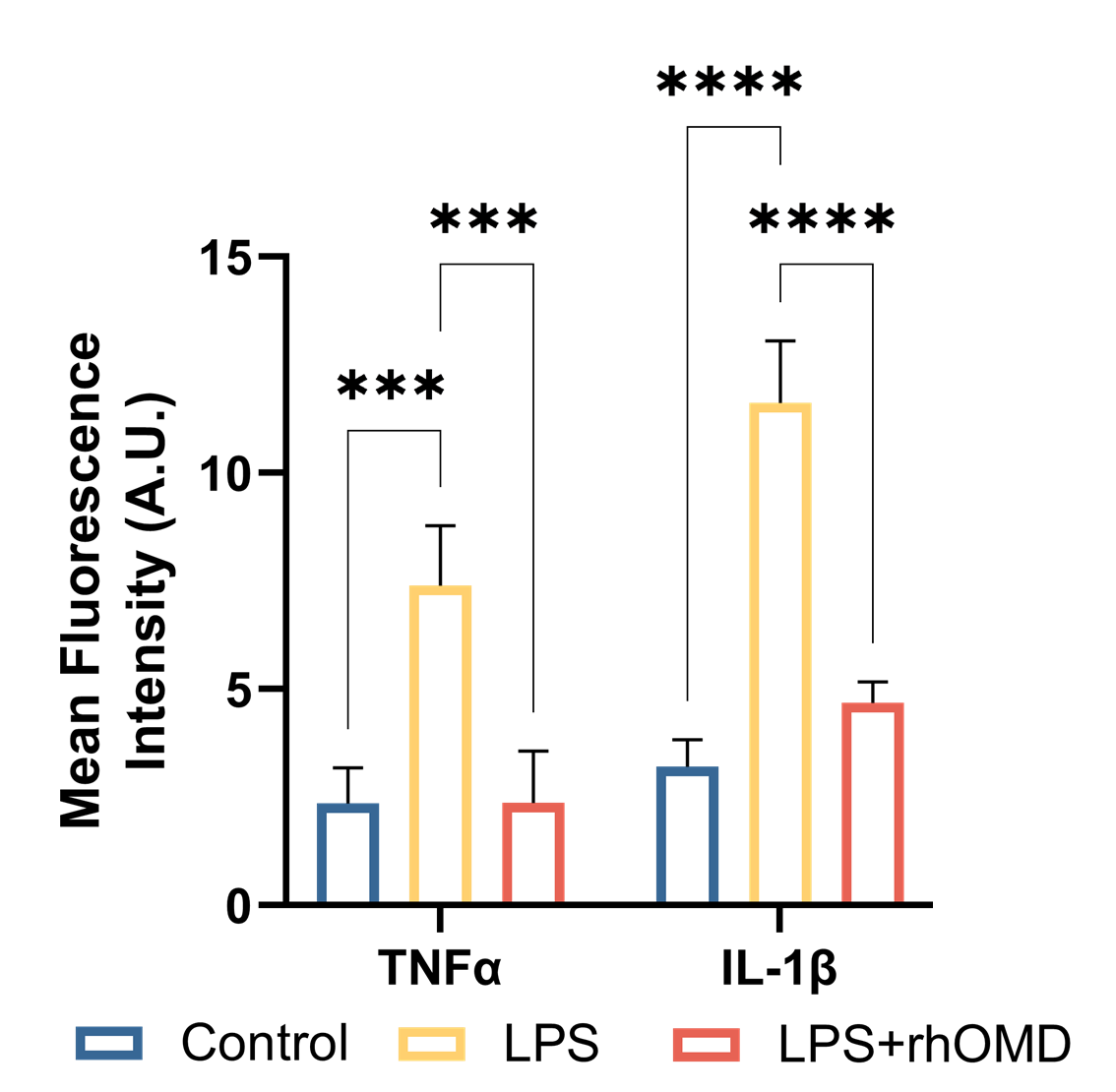
**

**Supplementary Figure 2.** Quantitative analysis of the red fluorescence intensity in (Fig 2e and 2f) using ImageJ. The results are expressed as mean ± SD (***P < 0.001 and ****P < 0.0001).

**
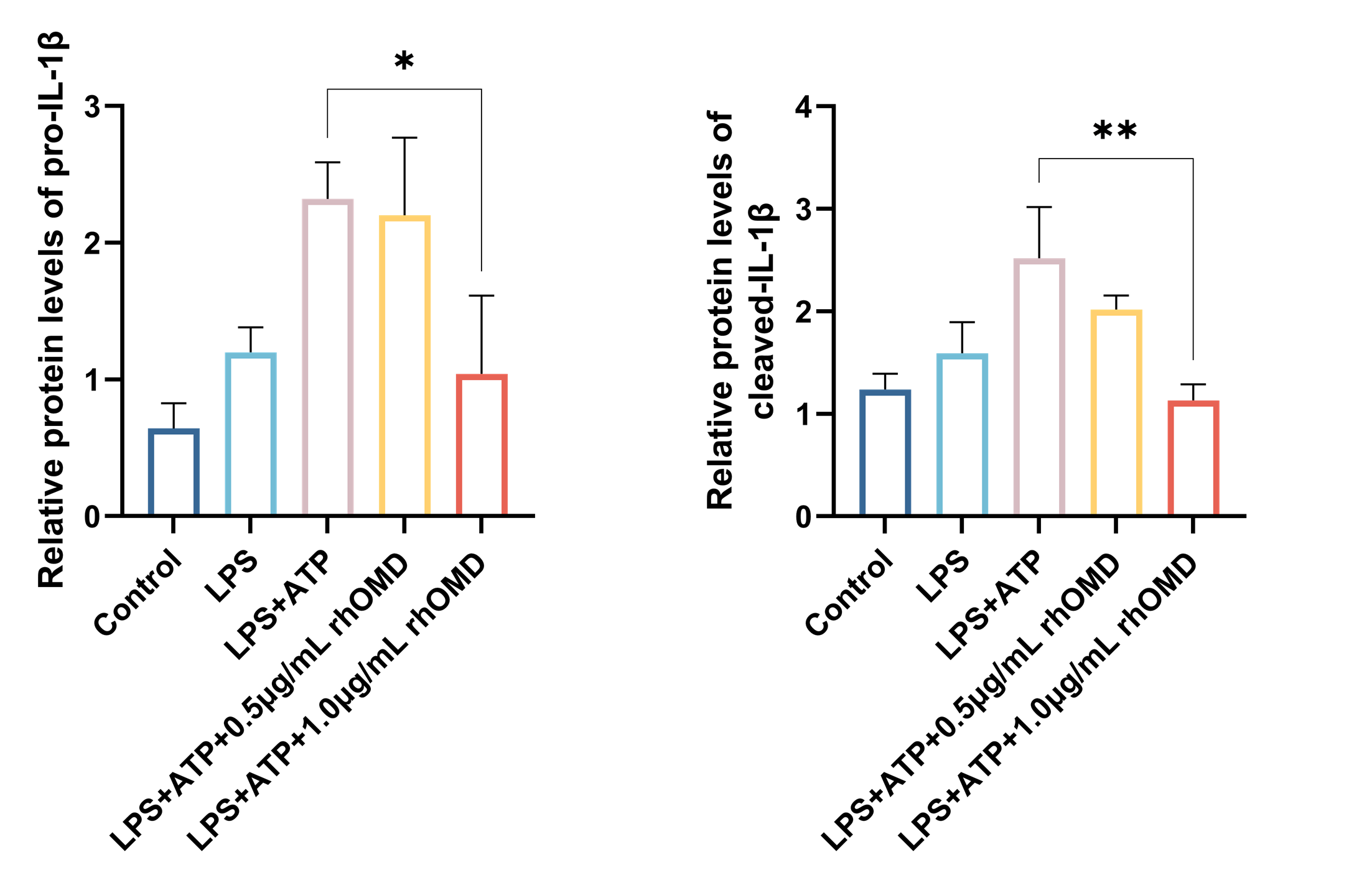
**

**Supplementary Figure 3.** Quantitative analysis of the relative protein levels of pro-IL-1β and cleaved-IL-1β in Figure 3d. The results are expressed as mean ± SD (^*^*P* < 0.05 and ^**^*P* < 0.01).

**
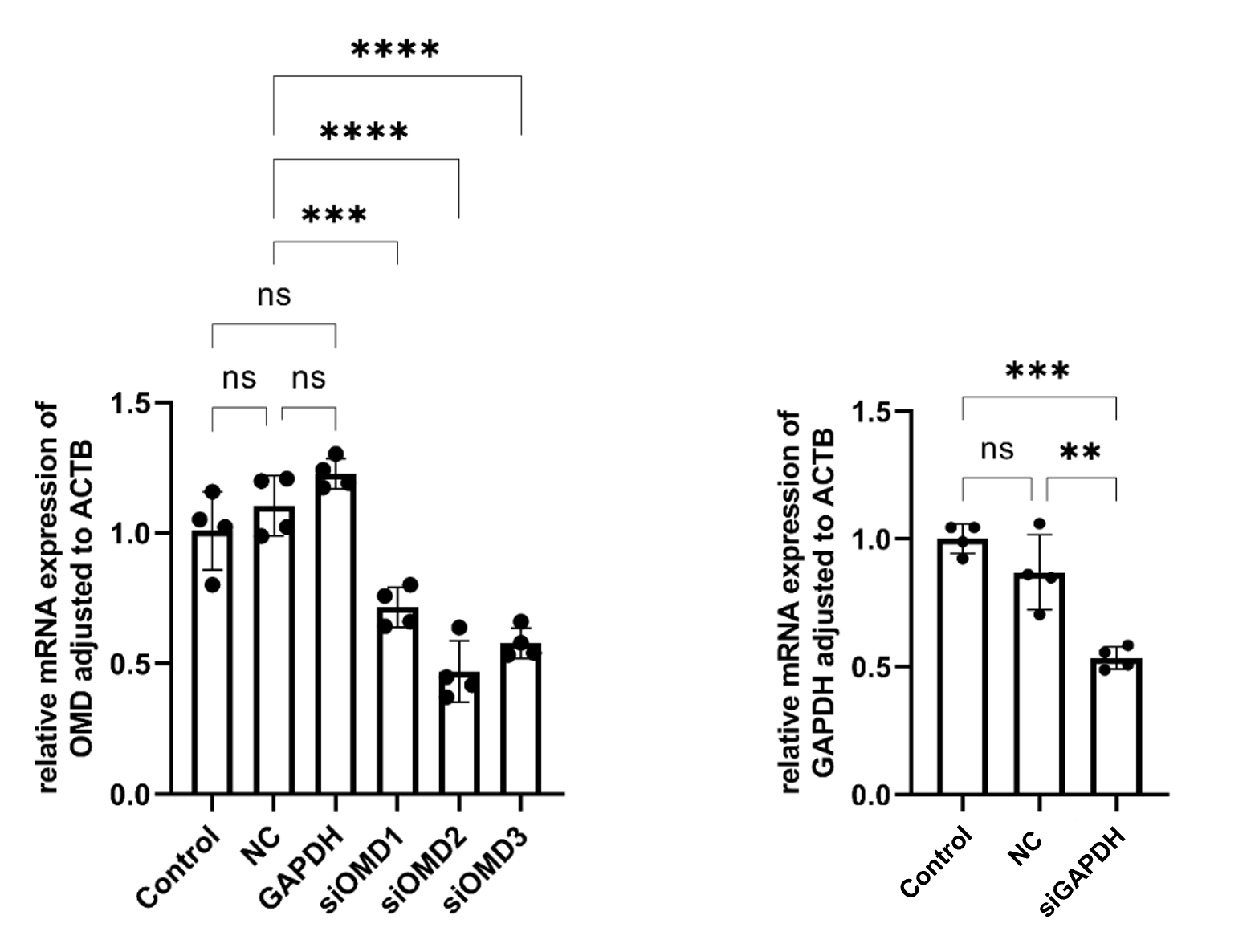
**

**Supplementary Figure 4.** RT-qPCR results showing the knockdown efficiency of siOMD for selecting the optimized siRNA. siGAPDH was used as a positive control. Results are expressed as mean ± SD (^**^*P* < 0.01, ^***^*P* < 0.001, and ^****^*P* < 0.0001; ns means no significance).


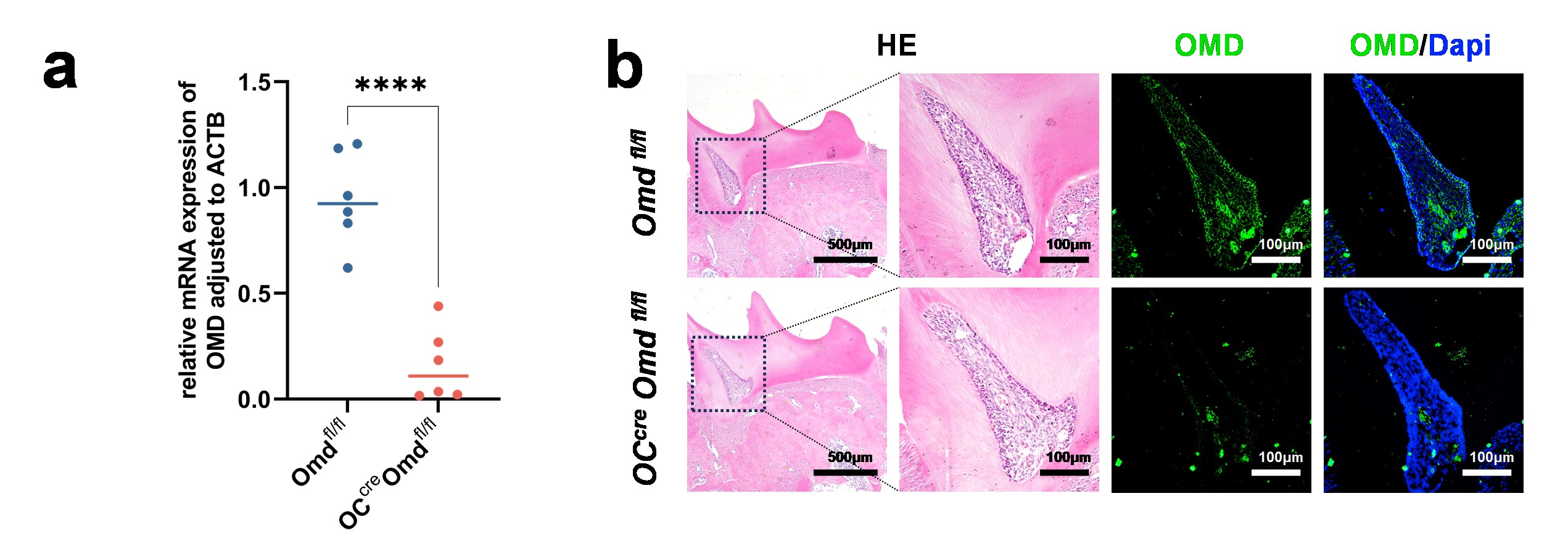


**Supplementary Figure 5.** (a) mRNA expression of *Omd* in the maxillary bone tissues of *Omd^fl/fl^* mice and *OC-Cre*; *Omd*^fl/fl^ mice (*n* = 6). (b) HE and immunofluorescence staining of OMD, with 5× magnification (scale bar: 500 μm) and 20× magnification (scale bar: 100 μm). Results are expressed as mean ± SD (^****^*P* < 0.0001).

**
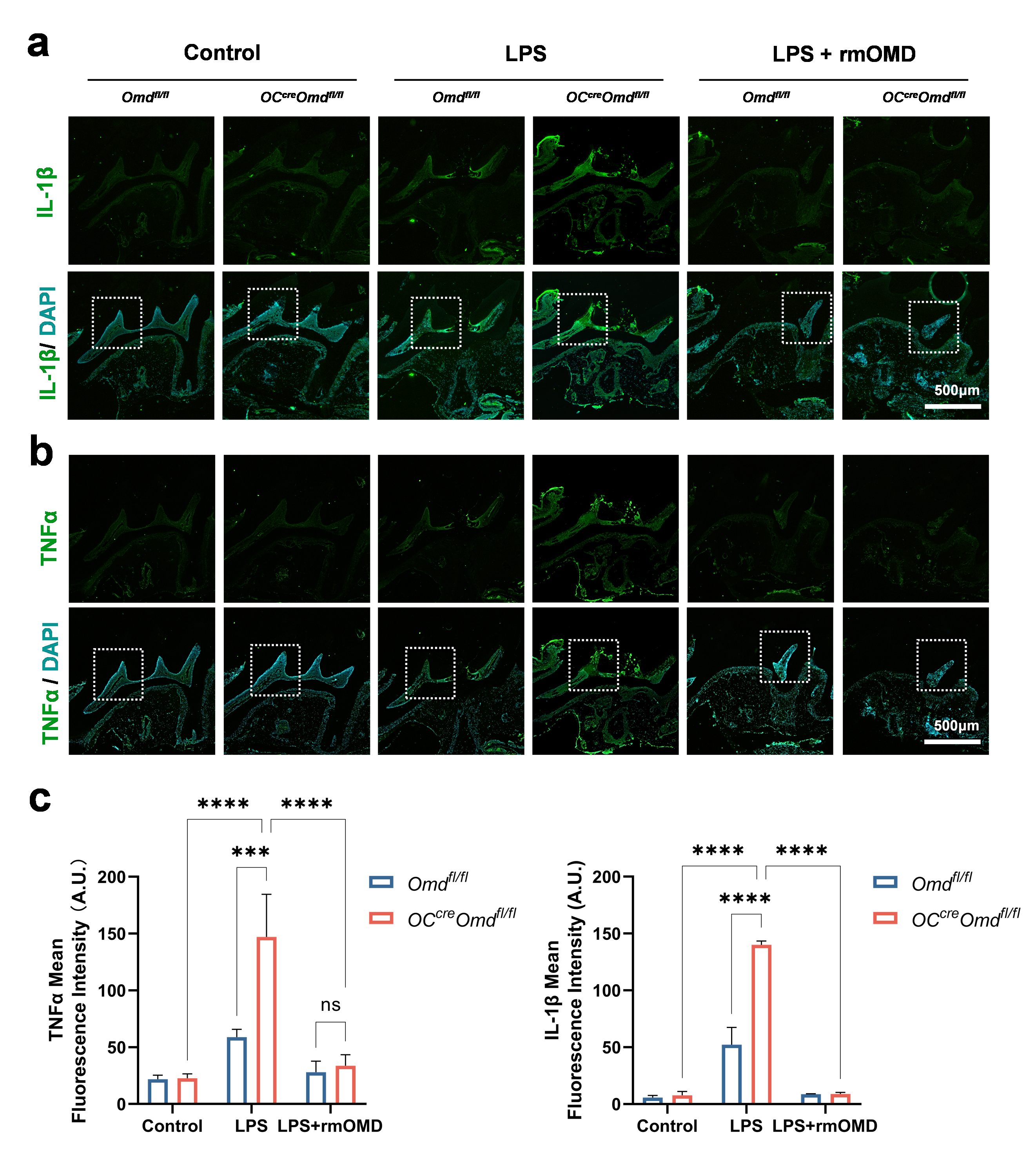
**

**Supplementary Figure 6.** (a, b) Immunofluorescence staining of TNFα and IL-1β, with 5× magnification (scale bar: 500 μm) (c) Mean immunofluorescence intensity of TNFα and IL-1β expression in (a). Results are expressed as mean ± SD (^***^*P* < 0.001 and ^****^*P* < 0.0001).

**
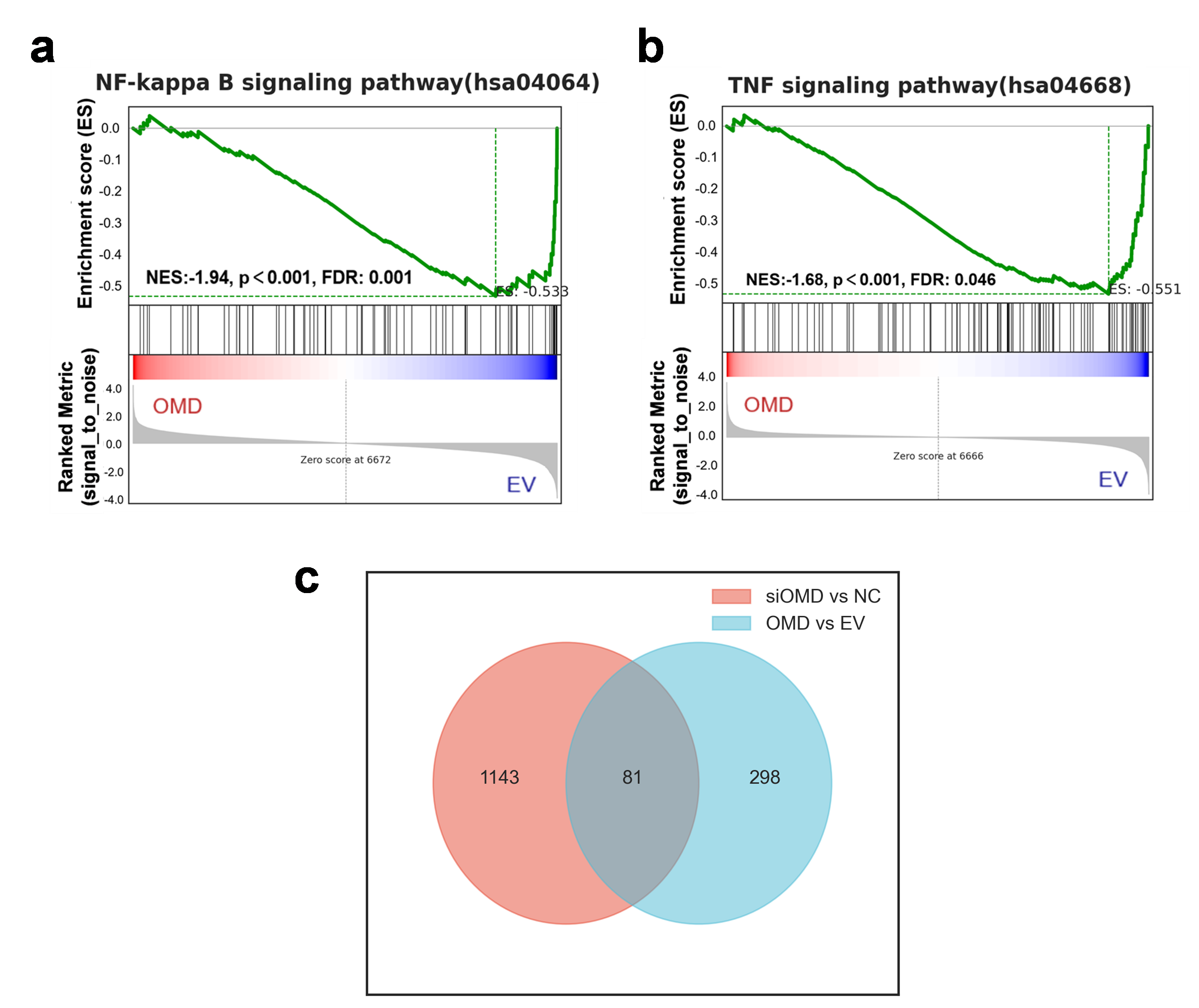
**

**Supplementary Figure 7.** (a, b) GSEA of NF-κB and TNF signaling pathways. (c) Venn diagram visualizing the number of co-occurring differentially expressed genes in the OMD-overexpressing hDPSCs and OMD-knockdown hDPSCs.


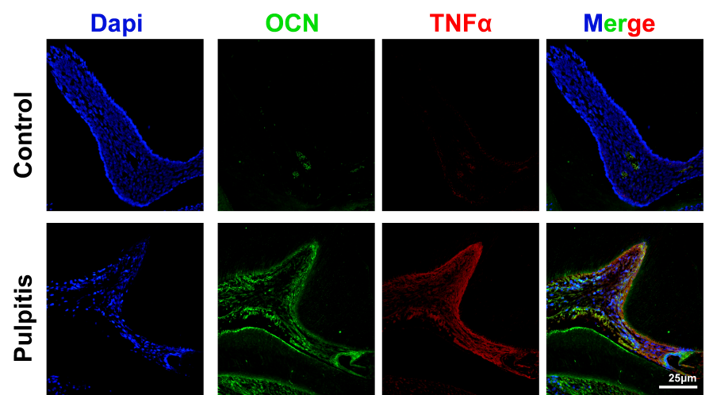


**Supplementary Figure 8.** Immunofluorescence staining of OCN and TNFα in normal pulp tissue and inflamed pulp tissue in mice.
